# Pre-fusion F is absent on the surface of formalin-inactivated respiratory syncytial virus

**DOI:** 10.1038/srep34108

**Published:** 2016-09-29

**Authors:** April M. Killikelly, Masaru Kanekiyo, Barney S. Graham

**Affiliations:** 1 Vaccine Research Center, National Institute of Infectious Diseases, National Institutes of Health, Bethesda, MD 20892, United States

## Abstract

The lack of a licensed vaccine for respiratory syncytial virus (RSV) can be partly attributed to regulatory hurdles resulting from vaccine enhanced respiratory disease (ERD) subsequent to natural RSV infection that was observed in clinical trials of formalin-inactivated RSV (FI-RSV) in antigen-naïve infants. To develop an effective vaccine that does not enhance RSV illness, it is important to understand how formalin and heat inactivation affected the antigenicity and immunogenicity of FI-RSV compared to native virus. Informed by atomic structures of RSV fusion (F) glycoprotein in prefusion (pre-F) and postfusion (post-F) conformations, we demonstrate that FI-RSV predominately presents post-F on the virion surface, whereas infectious RSV presents both pre-F and post-F conformations. This significant antigenic distinction has not been previously appreciated. Thus, a stabilized pre-F antigen is more representative of live RSV than F in its post-F conformation, as displayed on the surface of FI-RSV. This finding has major implications for discriminating current pre-F-based immunogens from FI-RSV used in historical vaccine trials.

Respiratory syncytial virus (RSV) is an enveloped, non-segmented negative-sense, single-stranded RNA virus that causes upper and lower respiratory tract infections. Nearly everyone is infected with the virus in the first two years of life; while reinfections occur throughout life, disease severity is highest in infants and the elderly. RSV represents a serious health and economic burden, and is the leading cause of hospitalization in children under the age of 5 (ref. 1).

Despite RSV being discovered nearly 60 years ago, no licensed vaccine is yet available. In part, this delayed development stems from clinical trials using formalin-inactivated RSV (FI-RSV) product that caused an enhanced respiratory disease (ERD) syndrome in children who received the FI-RSV vaccine. The FI-RSV vaccine adjuvanted with alum was evaluated in four separate studies in seronegative infants and young children in 1966 (refs 2, 3, 4, 5, 6). Instead of eliciting protective immunity, a greater number of vaccinees developed severe illness compared to control groups. A three dose regimen (0, 1, 4 months) was used in subjects between 2 and 7 months of age, 16 were hospitalized of the 20 infected children in the FI-RSV-vaccinated group (N = 31) compared to only 1 hospitalized of 21 infected in the control groups (N = 40)^3^. Tragically, two of the FI-RSV recipients died at 14 and 16 months of age from bacterial pneumonia complicating their subsequent RSV infection. In the majority of vaccinees, priming with FI-RSV led to pathology upon subsequent RSV infection that ordinarily is only manifest in a small fraction of RSV-naïve individuals. The immunological basis for FI-RSV-induced enhanced illness has focused on two major features of the humoral and cellular responses. First, FI-RSV induced high titers of binding antibody with weak neutralizing and fusion-inhibitory activity^7^,^8^. These antibodies in the context of large antigen load led to immune complex deposition and complement activation in airways upon subsequent RSV infection^9^. Second, natural RSV infection after immunization with FI-RSV was associated with exaggerated peribronchiolar inflammation and infiltration of neutrophils and eosinophils into airways. This is consistent with findings in animal models where FI-RSV has been shown to induce Th2-biased immune responses and airway hypersensitivity characterized by up regulation of IL-4, IL-5, IL-13, and IgE^10^.

Importantly, FI-RSV vaccination does not result in enhanced RSV disease when individuals are first primed with live virus infection or attenuated replication-competent vaccines are given intranasally or parenterally^11^,^12^, indicating that immunological priming with the FI-RSV vaccine was responsible for aberrant responses to subsequent infection. Therefore, to develop an effective vaccine that does not enhance RSV illness upon subsequent infection in antigen-naïve young infants, it is important to understand how viral inactivation affected antigenicity and immunogenicity of FI-RSV compared to native virus.

Formalin (aqueous formaldehyde) treatment is a long-established method to inactivate viruses. At high concentrations (1%), formalin fixes tissue and obliterates infectivity by forming chemical additions (carbonyls) and intra- and inter-protein crosslinking^13^. At lower concentrations, however, these modifications have a varied effect on preserving antigenic sites. Hepatitis A virus vaccine that was inactivated with 0.0625% formalin elicited antibodies in humans that neutralized virus and protected against infection^14^,^15^. However, poliovirus that was inactivated with 0.025% formalin was shown to have decreased binding to the human poliovirus receptor, suggesting that formalin inactivation had altered the receptor-binding site^16^. These data suggest that formalin can potentially “fix” or stabilize protein conformation, alter protein structure, and/or chemically modify protein surfaces. Until now it was unknown how the heat and formalin treatment used to produce FI-RSV would affect the conformation and antigenic content of the RSV fusion glycoprotein (F) on virion surfaces.

The surface of RSV is decorated with surface proteins, including the fusion protein (F), the attachment protein (G) and the small hydrophobic (SH) pentameric ion channel. While G may play a role in attachment to airway epithelium and immunomodulation, the F glycoprotein is the major target for vaccine development due to its requirement for viral entry and high sequence conservation. F is a type I transmembrane protein cleaved by furin-like proteases into F1 (AA 137–574), peptide 27 (AA 110–138) and F2 (AA 1–109) that form a trimer of F1-F2 heterodimers. Atomic-level structures of F have been determined in two distinct conformations, prefusion and postfusion^17^,^18^,^19^ (pre-F and post-F, respectively) (Fig. 1a,b). Similarly to other class I fusion proteins, RSV F undergoes a massive conformational rearrangement to mediate viral-cell membrane fusion, transitioning irreversibly from a metastable pre-F to a stable post-F conformation. Although pre-F and post-F structures have distinct shapes, they share roughly 50% of their surface area, including two well-defined antigenic sites (II and IV) that are moderately neutralization sensitive. On the unique surfaces of pre-F there are at least three additional antigenic sites reported so far (Ø, III, and V) that are highly neutralization sensitive, particularly site Ø (Fig. 1b). Antibodies that bind antigenic site I have weak or no neutralizing activity and bind almost exclusively to post-F. Both pre-F and post-F are present in the membrane of infectious RSV, but neutralizing antibody responses found in naturally infected individuals > 5 years of age are predominantly directed towards pre-F surfaces^20^. To have significant neutralizing activity, RSV F-specific antibodies have to recognize and interrupt pre-F function^21^.

At the time of FI-RSV production and clinical evaluation, it was thought that although formalin treatment would render RSV non-infectious, it would not interfere with the antigenic properties of the virus. Studies later showed that FI-RSV induced relatively high titres of binding antibodies, but with low neutralizing activity^7^,^8^, hinting that FI-RSV may not be recapitulating the antigenic surfaces of infectious RSV. Structural analyses detailing the epitopes of monoclonal antibodies (mAbs) binding to pre-F and post-F have yielded tools that make it possible to differentiate antigenic profiles of pre-F and post-F and determine the effect of inactivating processes (i.e.: heat and formalin) on the integrity of RSV F on the surface of FI-RSV in comparison with infectious virus.

## Results

**Figure 1:**
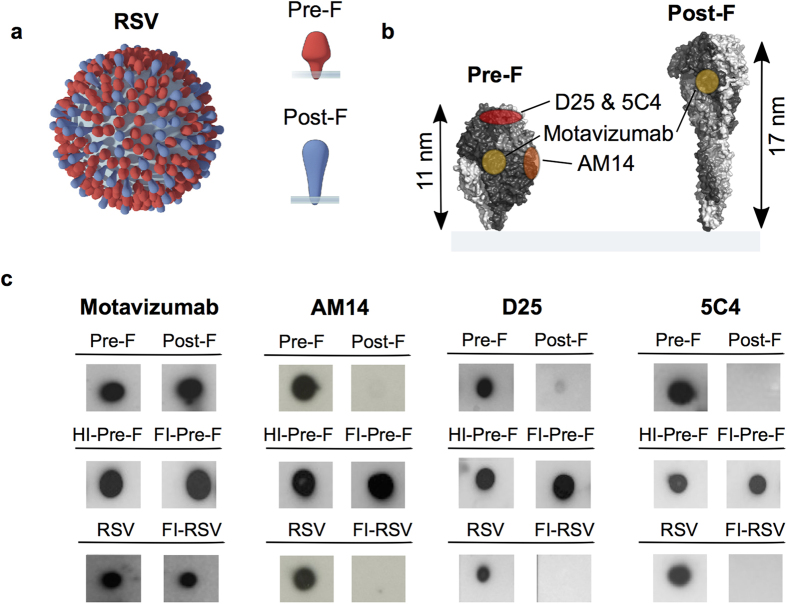
Antigenicity of RSV prefusion F and postfusion F. (**a**) Infectious respiratory syncytial viral particles display F surface glycoprotein in both pre-F and post-F conformations. (**b**) Surfaces of pre- and post-F (PDB IDs 4JHW and 3RRR, respectively) highlight each monomeric subunit of trimeric F in shades of grey. Representative neutralizing monoclonal antibodies targeting RSV F either bind to sites only on the surface of pre-F (AM14 (orange), D25 & 5C4 (red)) or to sites present on the surfaces of both pre- and post-F (Motavizumab (yellow)). (**c**) Dots of pre-F, post-F, Heat Inactivated (HI-) pre-F, Formalin Inactivated (FI-) pre-F, RSV or FI-RSV are applied to a nitrocellulose membrane and blotted with antibodies motavizumab, AM14, D25 and 5C4 similarly to a Western blot (Nadala 1999).

**Figure 2:**
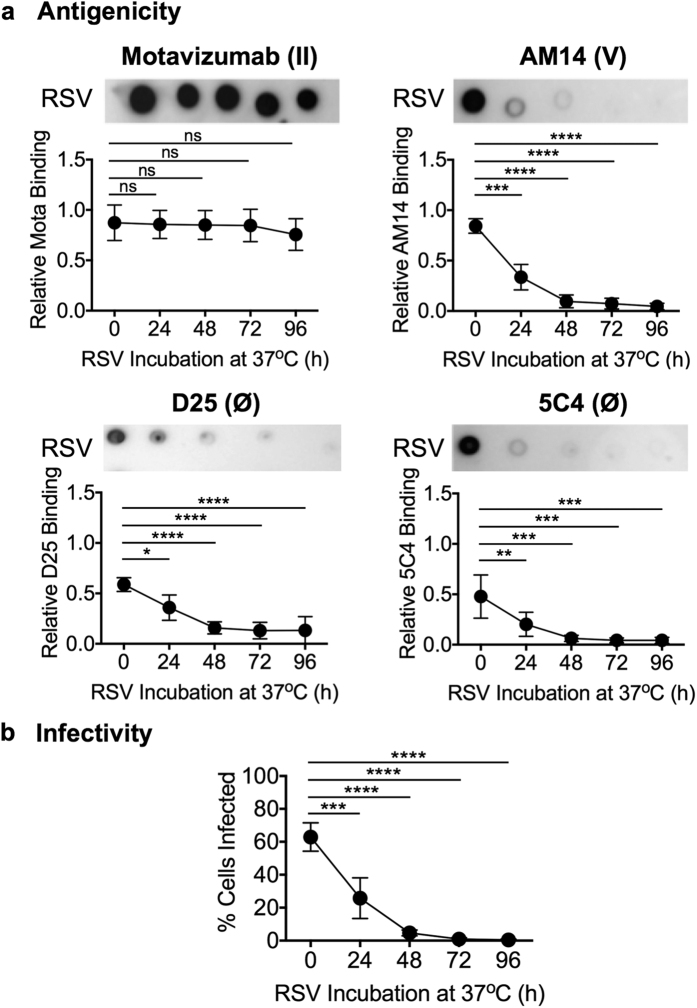
Assessing the stability of pre-F during FI-RSV production. (**a**) Dot blots demonstrate motavizumab recognition of antigenic site II, AM14 recognition of quaternary antigenic site V, and D25 and 5C4 recognition of antigenic site Ø after RSV is incubated at 37 °C with a 0.025% concentration of formalin in 24-hour intervals. Average antibody binding of virions is reported relative to antibody binding of a pre-F protein loading control with error bars representing standard deviation of three experiments. Significant differences between populations are calculated by One-Way ANOVA and noted graphically (ns, not significant; ^*^p ≤ 0.05; ^**^p ≤ 0.01; ^***^p ≤ 0.001; ^****^p ≤ 0.0001). Recognition of antigenic site II, present on both pre-F and post-F surfaces, is preserved after treatment with high temperature and a dilute concentration of formalin. In contrast, dot blots demonstrate pre-F-specific mAbs AM14, D25 and 5C4 rapidly lose reactivity against virions incubated at 37 °C with a dilute concentration of formalin. (**b**) Percentage of cells infected by RSV incubated at 37 °C with a 0.025% concentration formalin in 24-hour intervals by flow cytometry (Chen 2010).

Antigenic characterization by dot blot using mAbs that either target antigenic site II on the shared surface between pre-F and post-F (motavizumab) or epitopes unique to pre-F (D25, 5C4, and AM14)^21^,^22^ (Fig. 1b) showed that all four antibodies recognized freshly purified RSV, but FI-RSV was only recognized by motavizumab (Fig. 1c). In the experiments using a conformationally stabilized prefusion F protein (DSCav1), pre-F specific antibodies were able to recognize DSCav1 after incubation at 37 °C with or without the addition of 0.025% formalin. This indicates that the loss of recognition of FI-RSV by pre-F specific antibodies is not due to chemical modifications by formalin, but rather due to modifications of pre-F conformation. To investigate the effect of formalin on antigenic recognition, we increased the concentration of added formalin to 1% and found that the recognition of post-F by motavizumab and DSCav1 by all antibodies were greatly reduced (Supplementary Fig. 1). This indicates that formalin can influence antigenicity, but the effect is not discernable at the “formalin-inactivating” concentration of 0.025%, and is much less than the impact of temperature. To further understand the effect of formalin and elevated temperature on the antigenic profile of RSV virions, we recapitulated the methods used to manufacture FI-RSV and defined the kinetics of antibody reactivity. Immediately after the application of formalin, RSV retained both pre-F and post-F antigenic sites; however, over time at 37 °C, pre-F specific epitopes on the surface of RSV disappear (Fig. 2a). The disappearance of pre-F specific epitopes (particularly site Ø) correlated with reduction in infectivity, as determined by flow cytometry of recombinant GFP-expressing RSV-infected cells (Fig. 2b).

**Figure 3:**
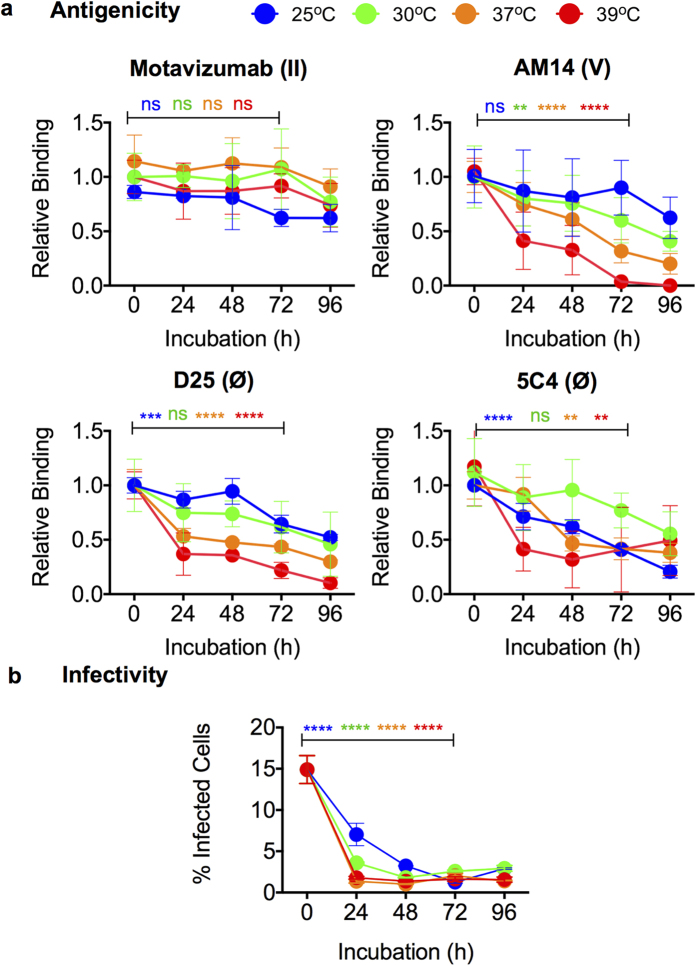
Temperature dependence of RSV inactivation. (**a**) Dot blots demonstrate motavizumab recognition of antigenic site II, AM14 recognition of quaternary antigenic site V, and D25 and 5C4 recognition of antigenic site Ø after RSV is incubated with a 0.025% concentration of formalin in 24-hour intervals at 25 °C (blue), 30 °C (green), 37 °C (orange) and 39 °C (red). Average antibody binding of virions is reported relative to antibody binding of a pre-F protein loading control with error bars representing standard deviation of three experiments. Significant differences between populations incubated for 0 and 72 hours are calculated by One-Way ANOVA and noted graphically (ns, not significant; ^*^p ≤ 0.05; ^**^p ≤ 0.01; ^***^p ≤ 0.001; ^****^p ≤ 0.0001). Recognition of antigenic site II, present on both pre-F and post-F surfaces, is preserved after treatment with a dilute concentration of formalin at all temperatures tested. In contrast, dot blots demonstrate a temperature dependent loss of pre-F-specific mAbs AM14, D25 and 5C4 lose reactivity against virions at every temperature tested. (**b**) Percentage of cells infected by virus incubated with 0.025% concentration of formalin in 24-hour intervals at 4 different temperatures as in (**a**) by flow cytometry (Chen 2010).

A similar correlation of diminished AM14 trimer binding and loss of infectivity was observed for virus incubated at different temperatures ranging from 25 °C to 37 °C in the presence of 0.025% formalin (Fig. 3a,b). While reactivity with motavizumab remains relatively stable at every temperature, virus incubated at close to room temperature (25 °C) maintains pre-F epitopes (AM14, D25, 5C4 binding) longer than viruses incubated at human body temperature (37 °C) or mouse body temperature (39 °C). These data indicate that pre-F on the surface of virions is converted to post-F more quickly at temperatures above room temperature; and that after 72 hours at 36 °C, the original FI-RSV had no remaining pre-F.

## Discussion

As a class I fusion protein, RSV F shares properties with surface glycoproteins of other enveloped viruses such as parainfluenza virus fusion (F), influenza hemagglutinin (HA), HIV-1 envelope (Env), Ebola glycoprotein (GP) and coronavirus spike (S). These proteins all mediate membrane fusion by a conformational change from a prefusion form to a transitional pre-hairpin intermediate form and eventually to a stable 6-helix bundle postfusion form. Therefore, defining the protein conformation presented on the surface of a viral immunogen is key to understanding mechanisms of antibody neutralization, and represents an important step toward designing a vaccine immunogen targeting the functional prefusion conformation. Recent work on HIV-1 Env has demonstrated that, in contrast to strain-specific antibodies raised against gp120 monomers or non-native trimeric gp140, stabilized versions of the prefusion Env trimer can preferentially elicit conformation-dependent, cross-reactive antibodies that can effectively target a functional prefusion conformation^23^. Similarly, the critical differences between antigenic sites of pre-F on infectious RSV and post-F on FI-RSV suggest that the balance of F conformational states determines virus infectivity. Designing immunogens that preserve neutralization-sensitive epitopes on the functional form of viral proteins will promote the induction of protective antibody responses and diminish non-neutralizing antibody responses, theoretically reducing the likelihood of immune complex formation and complement activation.

**Figure 4:**
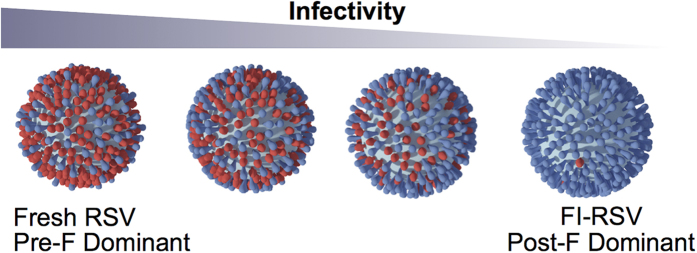
Graphic representation of parallel loss of infectivity and pre-F antigenicity as virions are treated to produce FI-RSV.

Studies in mice and macaques have shown that stabilized pre-F immunogens elicit potent neutralizing antibody responses with titers at the high end of what is achieved after recurrent natural infections in humans^21^. By 72 hours, the time used to make the FI-RSV vaccine, the presence of pre-F-specific epitopes on the virion surface of the membrane was nearly completely lost (Fig. 4). This contradicts the original presumption that production of FI-RSV from RSV alters only the infectivity of the virus, not the antigenicity, and that this low concentration of formalin would help preserve or fix the antigenic sites on the virus^24^.

The catastrophic failure and tragedy resulting from the FI-RSV vaccine trails has restricted consideration for using protein-based RSV vaccines in antigen-naïve infants. Defining the atomic-level detail of RSV F in two distinct conformations as well as isolation and characterization of mAbs that can distinguish surfaces unique to the functional pre-F from those on non-functional post-F, provides new reagents to define the antigenicity of FI-RSV and an additional piece of the FI-RSV-induced vaccine-enhanced disease puzzle. We anticipate that vaccine design based on understanding the relationship between conformational state and specificity of immune responses will guide the development of effective vaccines against RSV and potentially other viral diseases.

## Materials and Methods

### Antigenic Analysis

Antigenic characterization was performed by dot blot using mAbs that either target antigenic site II on the shared surface between pre-F and post-F (motavizumab) or epitopes unique to pre-F surfaces (D25, 5C4, and AM14)^21^,^22^. Briefly, dots of pre-F, post-F, heat inactivated pre-F (HI-Pre-F), formalin inactivated pre-F (FI-Pre-F), RSV or FI-RSV are applied to a nitrocellulose membrane, probed with antibodies motavizumab, AM14, D25 and 5C4 (made in house) similarly to a Western blot and probed with an HRP-conjugated secondary antibody reactive against either human (goat anti-human IgG-HRP, Santa Cruz Biotechnology) or mouse (rabbit anti-mouse IgG-HRP, Jackson Immunoresearch) Fc regions^25^. Both HI- and FI-Pre-F are incubated at 37 °C for 96 hours, FI-Pre-F includes a 0.025% concentration of formalin and 1% Pre-F includes a 1% concentration of formalin. Antibodies are incubated for one hour at room temperature in 5% skim milk in TBS-T at a concentration of 0.2 μg/mL. Antibody binding is detected via a G-box gel imager (Syngene) and densitometry done using Genetools (Syngene). Antibody binding to post-F and virions is reported relative to antibody binding of a pre-F protein loading control.

### Time Course Analysis

The manufacture of FI-RSV required RSV to be exposed to a 0.025% formalin for 72 hours at 36 °C. We produced FI-RSV by incubating RSV with 0.025% formalin at 37 °C for up to 96 hours. Viruses are sampled for antigenic analysis and infectivity every 24 hours over a 96 hour-interval after incubation at 25 °C, 30 °C, 37 °C or 39 °C.

### Infectivity

RSV expressing GFP (A2 strain) was incubated at 37 °C with a 0.025% concentration formalin and sampled periodically to infect HEp-2 cells^26^. Infection was monitored as a function of GFP expression (encoded by the viral genome) at 18 hours post- infection by flow cytometry (LSR II, BD Bioscience, CA, USA). Prior to assessment by flow cytometry, cells were treated with 4 mM EDTA to ensure a single-cell suspension optimal for analysis and fixed with 0.5% paraformaldehyde. One hundred percent infectivity represents the number of cells infected by RSV with no prior incubation.

## Additional Information

**How to cite this article**: Killikelly, A. M. *et al*. Pre-fusion F is absent on the surface of formalin-inactivated respiratory syncytial virus. *Sci. Rep.* **6**, 34108; doi: 10.1038/srep34108 (2016).

## Supplementary Material

Supplementary Information
