# Supplementary material for: Pre-fusion F is absent on the surface of formalin-inactivated respiratory syncytial virus
Source: Sci Rep. 2016 Sep 29;6:34108. doi: 10.1038/srep34108 (PMC5040956; doi:10.1038/srep34108)
Supplement: Supplementary Information [file srep34108-s1.pdf]

Supplementary Figure 1. High concentrations of formalin influence antigenicity of RSV pre-F and post-F. Boxes represent the blot cut-outs shown in Figure 1. Left, dots of pre-F, post-F, RSV or FI-RSV are applied to a nitrocellulose membrane and blotted with antibodies motavizumab, AM14, D25 and 5C4. Right, dots of pre-F and post-F incubated at 37ºC for 72 hours without formalin (heat-Inactivated, HI), with 0.025% formalin (formalin-Inactivated, FI), and with 1% formalin (1%) are applied to nitrocellulose membranes and blotted with antibodies motavizumab, AM14, D25 and 5C4 as above. Motavizumab is able to detect RSV, FI-RSV, post-F incubated with all concentrations of formalin, and pre-F incubated with 0.025% formalin or no formalin. AM14, D25 and 5C4 are able to detect only RSV (not FI-RSV) and only pre-F (not post-F) incubated with 0.025% formalin or no formalin. Neither AM14, D25 nor 5C4 are able to detect pre-F or post-F incubated at 1% formalin.

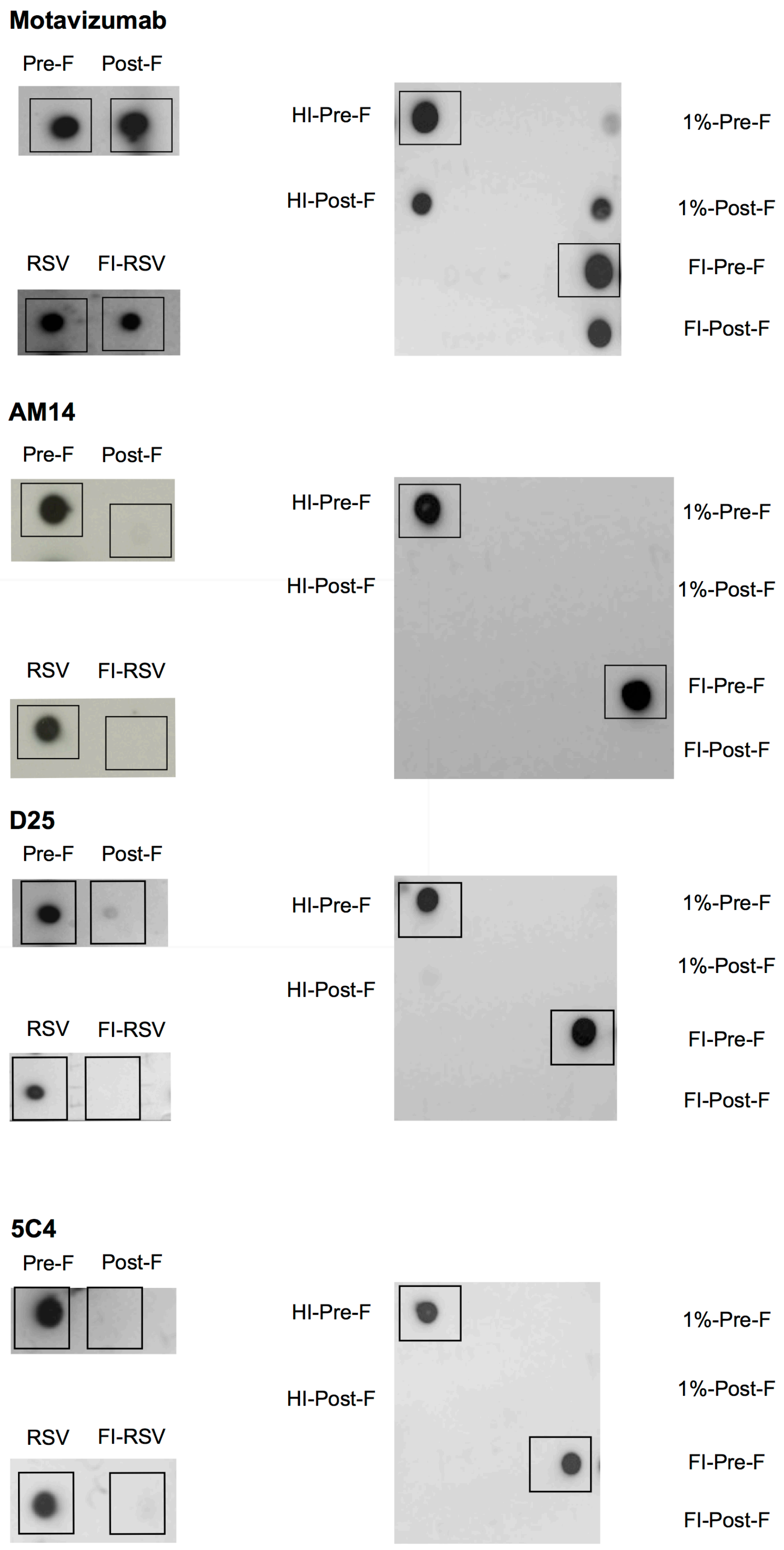
Motavizumab
Pre-F
Post-F
RSV
FI-RSV
HI-Pre-F
HI-Post-F
1%-Pre-F
1%-Post-F
FI-Pre-F
FI-Post-F
AM14
Pre-F
Post-F
RSV
FI-RSV
HI-Pre-F
HI-Post-F
1%-Pre-F
1%-Post-F
FI-Pre-F
FI-Post-F
D25
Pre-F
Post-F
RSV
FI-RSV
HI-Pre-F
HI-Post-F
1%-Pre-F
1%-Post-F
FI-Pre-F
FI-Post-F
5C4
Pre-F
Post-F
RSV
FI-RSV
HI-Pre-F
HI-Post-F
1%-Pre-F
1%-Post-F
FI-Pre-F
FI-Post-F
